# Chemical Analysis of Astragali Complanati Semen and Its Hypocholesterolemic Effect Using Serum Metabolomics Based on Gas Chromatography-Mass Spectrometry

**DOI:** 10.3390/antiox6030057

**Published:** 2017-07-21

**Authors:** Tung Ting Sham, Huan Zhang, Daniel Kam Wah Mok, Shun Wan Chan, Jianhong Wu, Songyun Tang, Chi On Chan

**Affiliations:** 1 Department of Applied Biology and Chemical Technology, The Hong Kong Polytechnic University, Hong Kong, China; irene.sham@connect.polyu.hk (T.T.S.); huanzi101@163.com (H.Z.); daniel.mok@polyu.edu.hk (D.K.W.M.); 2 State Key Laboratory of Chinese Medicine and Molecular Pharmacology (Incubation), Shenzhen 518057, China; 3 Food Safety and Technology Research Centre, Department of Applied Biology and Chemical Technology, The Hong Kong Polytechnic University, Hong Kong, China; 4 Department of Food and Health Sciences, Faculty of Science and Technology, Technological and Higher Education Institute of Hong Kong, Hong Kong, China; 5 Clinical Laboratory, Shenzhen Nanshan Center for Chronic Disease Control, Shenzhen 518000, China; wujianhong_sz@126.com; 6 The Center Hospital of Hengyang, Hengyang 421001, China; tangsy55@163.com

**Keywords:** Astragali Complanati Semen, serum metabolomics, hypercholesterolemia, mass spectrometry

## Abstract

The hypocholesterolemic protective effect of the dried seed of *Astragalus complanatus* (ACS) was investigated in rats fed with normal diet, high cholesterol diet (HCD), and HCD plus 70% ethanol extract of ACS (600 mg/kg/day) by oral gavage for four weeks. ACS extract was tested to be rich in antioxidants, which may be contributed to its high content of phenolic compounds. Consumption of ACS remarkably suppressed the elevated total cholesterol (*p* < 0.01) and LDL-C (*p* < 0.001) induced by HCD. Chemical constituents of ACS extract were analyzed by ultra-performance liquid chromatography coupled with electrospray ionization orbitrap mass spectrometry and the results showed that the ACS extract mainly consisted of phenolic compounds including flavonoids and flavonoid glycosides. In addition, based on the serum fatty acid profiles, elucidated using gas chromatography-mass spectrometry, free and esterified fatty acids including docosapentaenoic acid, adrenic acid, dihomo-γ-linolenic acid and arachidonic acid were regulated in ACS treatment group. Western blot results further indicated the protein expression of peroxisome proliferator-activated receptor alpha (PPARα) (*p* < 0.05) in liver was upregulated in ACS treatment group. To conclude, our results clearly demonstrated that ACS provides beneficial effect on lowering HCD associated detrimental change.

## 1. Introduction

Astragali Complanati Semen (ACS), alternatively known as Flatstem Milkvetch Seed or ShaYuanZi in Chinese, is the dried ripe seeds of *Astragalus complanatus* Bunge (*Leguminosae*) [1], recorded in Chinese Pharmacopoeia [2] and Hong Kong Materia Medica Standards [3]. It is commonly used by Chinese medicine practitioners to tonify the “kidney” and assisting “yang”, securing essence to reduce urination, and nourishing “liver” to improve eyesight [2]. ACS also possesses anti-hypertensive and anti-fibrosis activities. Its total flavonoids fraction has demonstrated anti-hypertensive action in rats via dilation of blood vessel by the blockade of the angiotensin II receptor [4] and anti-fibrotic action in rats associated with the regulation on lipid peroxidation and collagen synthesis [5]. Recently, the content of phenolic fraction in ACS has been shown to be highly correlated with the antioxidant activity [6]. It is believed that many of these therapeutic effects are attributed to its main constituents such as organic acids, flavonoids and its glycosides and triterpenoids.

Metabolomics aims at comprehensive characterization of endogenous metabolites and their variation in complex biological matrices such as plasma, urine and tissues extract by using high resolution nuclear magnetic resonance and mass spectrometry (MS) [7,8]. The methodology is particularly valuable for studying the systemic responses because of disease and drug treatment [9,10,11,12,13]. Thus, the use of metabolomics techniques should be a promising in understanding multi-component of herb extract in the treatment of metabolic diseases.

Hyperlipidemia is one of the major controllable risk factors for cardiovascular diseases (CVDs) [14]. It is characterized by high levels of one or more lipids and/or lipoproteins including atherogenic free fatty acids, triacylglycerols (TG), low density lipoprotein cholesterol (LDL-C), and apolipoprotein B, and/or low level in high density lipoprotein cholesterol (HDL-C) in blood and all those values have been remained as clinical indicator for CVD in the society. Investigation of fatty acid profile in circulation could be reflected by cellular lipid homeostasis, which is characterized by the balance between processes that generate or deliver fatty acids and processes that utilize these molecules [15]. This approach has been proposed and utilized to study the obesity and other related metabolic diseases [16,17,18].

In the present work, fatty acid profiling including esterified fatty acids (EFAs) and free fatty acids (FFAs) were examined to study the metabolite changes in the serum samples collected from rats and investigate the therapeutic effect of ACS on high cholesterol diet (HCD)-induced hypercholesterolemic rats. Gas chromatography-mass spectrometry (GC-MS) results indicated that ACS regulated several fatty acids in serum after treatment. It was further confirmed that the protein expression level of hepatic peroxisome proliferator-activator receptor α (PPARα) was upregulated by using Western blot. To the best of our knowledge, this is the first study demonstrating ACS supplement lowered circulating cholesterol levels, which was elevated due to HCD, by regulating the fatty acid metabolism via the metabolomics platform.

## 2. Materials and Methods

### 2.1. Chemical and Solvents

HPLC-graded acetonitrile, methanol and hexane were obtained from Fisher Scientific (Hampton, NH, USA). Absolute ethanol was purchased from VWR chemicals (Radnor, PA, USA). Potassium hydroxide (Guangzhou Chemical Reagent Factory, Guangzhou, China), sulfuric acid (>98% purity, Chengdu Kelong Chemical Reagent Company, Chengdu, China) with and anhydrous sodium sulfate (Tianjin Damao Chemical Reagent Factory, Tianjin, China) used in the fatty acid derivatization process were analytical grade. l-ascorbic acid, 2,2-diphenyl-2-picrylhydrazyl (DPPH), Folin–Ciocalteu reagent, sodium carbonate, analytical graded formic acid and gallic acid (98% purity), heptadecanoic acid (C17:0, 99.0% purity), heptadecanoic acid methyl ester (EC17:0, 99.0% purity), oleic acid (C18:1n-9, ≥99.0% purity), oleic acid methyl ester (EC18:1n-9, ≥99.0% purity) and 37 fatty acid methyl ester (FAME) standard mixture (FAME Mix 37, Supelco) were commercially obtained from Sigma-Aldrich company (St. Louis, MO, USA).

The following chemicals for ultra-performance liquid chromatography coupled with electrospray ionization orbitrap mass spectrometry (UPLC-ESI-Orbitrap-MS) identification were used: formononetin (LKT Laboratories, St. Paul, MN, USA), isoquercitrin (Shanghai Tauto Biotech, Shanghai, China), complanatuside (Chengdu Must Biotechnology, Chengdu, China), kaempferol and quercetin (International Laboratory, South San Francisco, CA, USA). Roche Reagents were obtained from Roche diagnostics (Mannheim, Germany). Simvastatin 20 mg tablets, a cholesterol-lowering drug, were purchased from Merck Sharp & Dohme (Hangzhou, China) as our positive control in the animal study. Water was purified by a Milli-Q water-purification system (Millipore, MA, USA).

### 2.2. Preparation of Extract of Astragali Complanati Semen (ACS)

**Figure 1:**
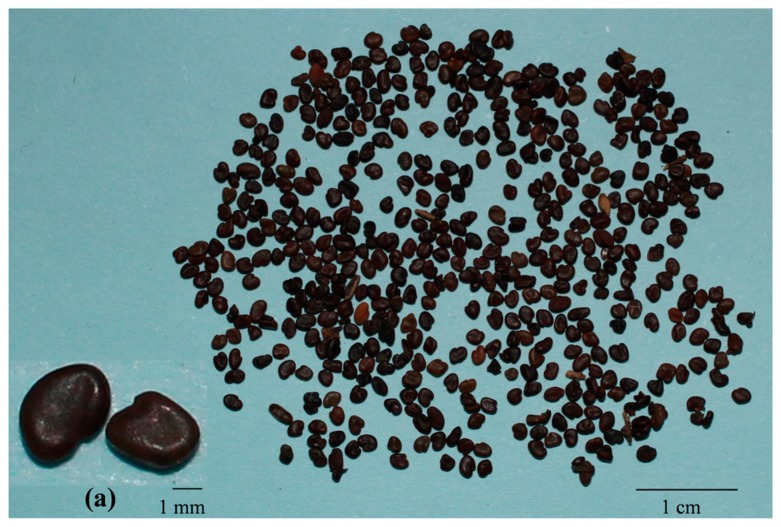
A photograph of Astragali Complanati Semen (ACS): (**a**) magnified ACS.

Ten batches of ACS were purchased from local market and Mainland China. The samples were authenticated by Dr. Sibao Chen based only on its morphological and microscopic characteristics [2]. All samples were then carried out fingerprint analysis based on Hong Kong Materia Medica Standards [3] and the obtained ten batches fingerprints were similar to each other. Figure 1 shows its morphological appearance of ACS used in this study (batch no: LSYZ-026-04) and its content of complanatuside was fulfilled the requirement of Chinese Pharmacopeia. ACS was ground into fine powder and mixed with 70% ethanol in a ratio 1:3 (*w*/*v*). The mixture was let stand for 1 h and then boiled under reflux for 1 h. Afterward, the supernatant was collected and the residue was re-extracted for two more times. Finally, supernatant was filtered. The filtrate was put in a rotary evaporator (Laborota4000, Heidolph, Germany) to remove the organic solvent at 50 °C water bath before lyophilizing in a freeze dryer (Labconco, Freezone 6, Kansas City, MO, USA). The extraction yield was 16.26%. The dried ACS extract in powder form was stored at −20 °C until it was redissolved in distilled water by ultrasonication before animal feeding.

### 2.3. Total Phenolic Quantification

The total phenolic content in the dried ACS extract was determined by the Folin–Ciocalteu method, in accordance to the procedure of Singleton [19]. Dried ACS extract was redissolved in water to make extract solution in three suitable concentrations (0.5, 0.8 and 1 mg/mL). As for the standard curve, different concentrations of gallic acid ranged from 0.01–0.08 mg/mL were freshly prepared. 500-µL Folin–Ciocalteu reagent was mixed with 400 µL of sodium carbonate (75.05 g/L) and then 100 µL of the extract solution or standard solution were added. After 2 h of incubation at room temperature, the absorbance of the samples and standard solution were measured at 750 nm with GENESYS™ 20 Visible Spectrophotometer (Thermo Fisher Scientific, West Palm Beach, FL, USA). Results were expressed as milligrams of gallic acid equivalents (GAE) per gram of dried ACS extract (mg GAE/g dried ACS extract). Content of total phenols of ACS was calculated using the following equation based on the calibration curve of absorbance against concentration of gallic acid: *y* = 19.18*x* + 0.0457 and *R*^2^ = 0.9937, where *y* was the absorbance and *x* was mg GAE/g dried ACS extract. The above process was repeated twice more. Data are presented as the mean ± standard error of mean (SEM).

### 2.4. DPPH Free Radical Scavenging Activity

DPPH is a stable free radical in red-purple colour that can be scavenged by antioxidants, resulting in discoloration and the reduction of absorbance at 515 nm. It has been widely applied to assess the antioxidative activities such as Chinese medicinal herbs [20,21]. The free radical scavenging activity (FRSA) of DPPH was measured by a modifcation from procedure described [22]. The FRSA of dried ACS extract was measured compared with vitamin C (l-ascorbic acid) as reference in parallel. Dried ACS extract redissolved in 70% ethanol by ultrasonication (0.02–10 mg/mL) and vitamin C in water (0.002–1 mg/mL) was freshly prepared. Fifty microliters of different concentrations of ACS extraction solution and vitamin C solution was diluted 20 times with DPPH methanol solution (24 mg/L). Blank solvents were prepared as control. After vortexing, the solutions were kept in the dark for 1 hour at room temperature. Two hundred microliters of each solution was aliquoted to three wells of the same 96-well plate. The absorbance of DPPH solution was obtained at 515 nm by a spectrophotometer CLARIOstar (BMG LABTECH, Ortenberg, Germany). The FRSA was determinded in terms of the scavenging activity in percentage (SR %) by the equation: SR% = (1 − A_sample_/A_control_) × 100% where A_sample_ and A_control_ were the absorbance with and without samples respectively. The half maximal effective concentration (EC_50_) of the samples were determined, which was the concentration of ACS or vitamin C to achieve 50% of scavenging capacity. The above test was repeated twice more to get an average of the capability to scavenge the DPPH radicals.

### 2.5. Chemical Analysis of ACS by Ultra-Performance Liquid Chromatography Coupled with Electrospray Ionization-Orbitrap-Mass Spectrometry (UPLC-ESI-Orbitrap-MS)

10 mg/mL of the dried ACS extract was prepared by redissolving 0.5 g of its extract with 50 mL 70% ethanol by ultrasonication for 20 min. Then, 2 mL extraction solution was centrifuged at 13,000× *g* for 15 min prior to UPLC-ESI-Orbitrap-MS analysis. A 3-µL aliquot was injected into a Waters ACQUITY UPLC system. The separation was performed on a Waters ACQUITY UPLC BEH C_18_ column (2.1 mm × 100 mm, 1.7 µm) with BEH C_18_ guard column (2.1 mm × 5 mm, 1.7 µm, Waters Corporation, Milford, MA, USA). The mobile phase consisted of combinations of A (0.1% formic acid in water, *v*/*v*) and B (0.1% formic acid in acetonitrile, *v*/*v*) at a flow rate of 0.3 mL/min with elution gradient as follows: 0 min, 10% B; 12 min, 40% B; 15 min, 60% B; 17–19.5 min, 95% B. A 3-min post-run time was set to fully equilibrate the column. Column and sample chamber temperature were 35 °C and 6 °C, respectively.

Mass spectrometry analysis was achieved by a Thermo Scientific Orbitrap Fusion Lumos Tribrid mass spectrometer equipped with a heated electrospray ionization (H-ESI) interface (Thermo Fisher, Waltham, MA, USA). The mass-spectrometric conditions were optimized as follows: spray voltage, 2.3 kV in negative H-ESI mode; ion transfer tube and vaporizer temperature, 300 °C. Nitrogen gas (≥99.999%) was used as the sheath gas and the aux gas with flow rate of 30 and 10 arbitrary unit respectively. The instrument was operated in data-dependent acquisition mode, with full MS scans over a mass range of m/z 100–1000 with detection in the Orbitrap (120k resolution) and with auto gain control (AGC) set to 80,000 and a maximum injection time at 100 ms. In each cycle (0.6 s) of data-dependent acquisition analysis, the most intense ions with intensity threshold above 20,000 were selected for fragmentation at normalized collision energy of 30 ± 10% higher energy collisional dissociation. The number of selected precursor ions for fragmentation was determined by the “Top Speed” acquisition algorithm. Fragment ion spectra were acquired in the Orbitrap (30k resolution) with an AGC of 20,000 and a maximum injection time of 35 ms for Orbitrap MS^2^ detection. All data analysis was carried out using Thermo Xcalibur Qual Browser software (Thermo Fisher Scientific).

### 2.6. Animals and Experimental Treatment

Thirty-two male Sprague Dawley rats (170 ± 10 g) supplied by Guangdong Provincial Medical Laboratory Animal Center (Guangzhou, China) were housed under specific pathogen free standard conditions (temperature at 24 ± 2 °C, humidity at 60 ± 10% and light from 6 a.m. to 6 p.m.) with free access to water and rat chow. After acclimation for a week in this housing environment, the rats were randomly divided into four groups (*n* = 8/group): (1) normal control: a control group fed with normal rat chow obtained from Guangdong Provincial Medical Laboratory Animal Center (Guangzhou, China) (rat chow composition: protein (~14%), fat (~10%), and carbohydrate (~76%)); (2) HCD model: a model group fed with HCD, which is the standard rat chow supplemented with 1% cholic acid, 2% pure cholesterol and 5.5% peanut oil; (3) positive control: a treatment group that received HCD plus simvastatin (3 mg/kg, body weight/day); (4) ACS: a study treatment group that received HCD plus ACS (600 mg/kg, body weight/day); The rats were administered with distilled water (vehicle) or their corresponding treatments by oral gavage once every morning for four weeks. At the end of the experimental period, the rats were fasted overnight and killed by cervical dislocation. Blood and livers were then collected for further analysis. The experimental protocol was conducted under the animal license issued by the Health Department of the Hong Kong SAR Government and the Animal Subjects Ethics Sub-committee (ASESC number 01/21) of the Hong Kong Polytechnic University (Hong Kong, China).

### 2.7. Analysis of Serum Lipid Profiles

Blood was collected in centrifuge tubes by cardiac puncture and allowed to clot for 2 h. Afterward, the clotted blood was centrifuged (3000× *g*) at 4 °C for 10 min to obtain the serum. Serum was stored at −80 °C freezer before measurement of lipid profiles. Content of total serum cholesterol, triacylglycerols, LDL-C, and HDL-C were determined by the ALYCON systems using Roche Reagents (Roche diagnostics, Mannheim, Germany). The atherogenic index was calculated by this equation: atherogenic index = (total serum cholesterol − HDL-C)/HDL-C.

### 2.8. Metabolomics Analysis

#### 2.8.1. GC-MS Sample Preparation Method

The methods reported [23] were used as a starting point for the method development. A two-step methylation of EFAs and FFAs fractions in each serum without a protein-removal step was applied. 200-μL serum aliquot was spiked with internal standards (20 μL, 3 mM EC17:0 and C17:0 in methanol). Trace amount of anhydrous sodium sulfate was added to remove water in the serum. In the first step, 2 mL of 0.4 M methanolic potassium hydroxide was added to the serum aliquot, vortexed for 1 min and let stand at room temperature for 20 min. Then, 2 mL *n*-hexane was added, vortexed for 30 s and the upper layer of hexane phase was isolated. The hexane extraction was done in twice and combined to isolate EFA methyl esters. In the second step, 2 mL 10% methanolic sulfuric acid was added to the serum phase and vortexed for 1 min. The mixture was incubated in a water bath at 70 °C for 30 min. Isolation of FFA methyl esters by hexane extraction was the same as EFA methyl esters. The two hexane phases of EFA and FFA methyl esters were dried with nitrogen gas separately. The dried sample was finally reconstituted with 1 mL *n*-hexane before GC-MS analysis.

#### 2.8.2. GC-MS Condition

The GC-MS system was an Agilent 6890N GC/5975C VL MSD system equipped with an Agilent 7683 Automatic Liquid Sampler (Agilent technologies, Inc., Santa Clara, CA, USA). The column was a DB-WAX column (30 m × 0.25 mm, 0.25 µm; Agilent J&W Scientific, Folsom, CA, USA). The inlet temperature of the GC was kept at 240 °C. Helium (≥99.999%) was used as carrier gas with a constant linear velocity of 1.0 mL/min. 1-μL aliquot was injected in splitless mode. The temperature program that was optimized for GC was as follows: the initial oven temperature as 70 °C, held for 1 min; 20 °C /min to 150 °C; 10 °C/min to 190 °C; 3 °C/min to 220 °C; 5 °C/min to 230 °C; 230 °C held for 12 min. The MS conditions were as follows: electron impact mode at ionization energy of 70 eV; ion source temperature at 230 °C; transfer line temperature at 240 °C; full scan mode in m/z range 35–550 with 0.3 s/scan velocity. The solvent delay was 3 min.

#### 2.8.3. Data Processing and Analysis

Peak identification of GC-MS data was carried out by comparing the retention time of authentic standards (37 FAME mix) and the mass spectra with NIST11 library. The peak detection and deconvolution were performed by Automated Mass Spectral Deconvolution and Identification System (AMDIS) v2.7 (NIST, Gaithersburg, MD, USA). Peak area was normalized by the spiked internal standard in each sample. Partial least squares discriminant analysis (PLS-DA) of the normalized peak area after pareto-scaling was performed by the Extended Statistical tool (EZinfo v2.0 software, Umetrics AB, Umeå, Sweden). Potential markers of interest were obtained based on their Variable Importance in the Projection (VIP) values of PLS-DA (threshold of VIP ≥ 1). 

### 2.9. Western Blot Immunoreactivity Assay

Hepatic peroxisome proliferator-activator receptor α (PPARα) and β-actin protein levels were quantified using immunoblotting procedures. Protein extracts (40 μg) of rat liver tissue were applied to 10% SDS polyacrylamide gels and transferred to polyvinylidene difluoride membranes, which were blotted overnight at 4 °C with non-fat milk solution. The membrane was probed with primary antibody (1:1000) followed by secondary antibody and visualized using an enhanced chemiluminescence (ECL) plus kit (Amersham Bioscience, Aylesbury, UK) and exposed to FujiFilm autoradiographic films according to the manufacturer’s protocol. Densitometric measurements of band intensity in the Western blots were performed using Quantity One Software (Bio-Rad, Hercules, CA, USA).

### 2.10. Statistical Analysis

Data were expressed as means ± standard error of mean (SEM) and n denotes the number of replications for each data point. Statistical differences of multiple groups were analyzed at a univariate level by one-way analysis of variance (ANOVA) followed by Tukey HSD post hoc test using GraphPad Prism 5.02 (San Diego, CA, USA). A value of probability *p* < 0.05 was considered statistically significant.

## 3. Results

### 3.1. Chemical Constituents of ACS Extract

**Figure 2:**
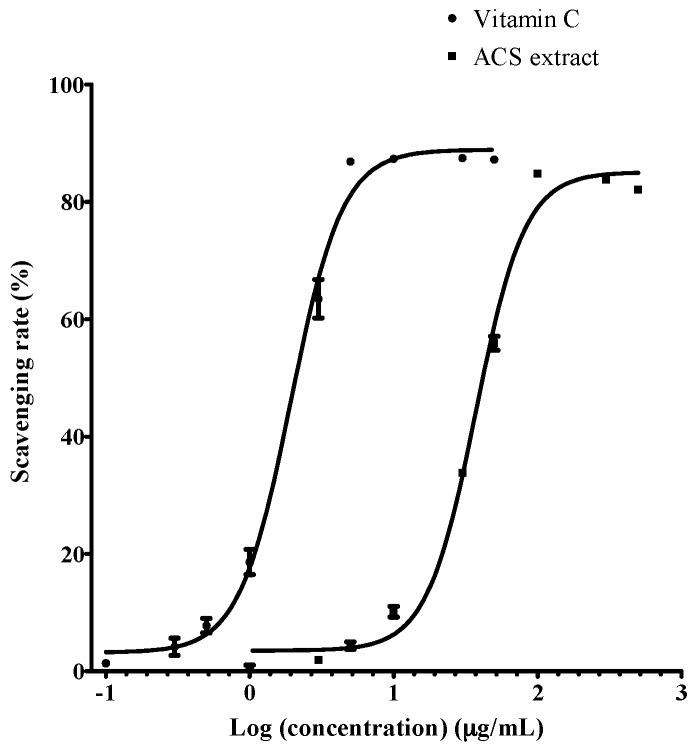
The free radical scavenging capacity of vitamin C and ACS against their concentration [mean ± standard error of mean (SEM), *n* = 3].

The total phenolic content of the dried ACS extract was determined to be 33.9 ± 0.7 mg GAE/g extract. ACS possessed high antioxidant property with EC_50_ = 37.30 ± 0.61 µg/mL by DPPH assay, with vitamin C (ascorbic acid, EC_50_ = 1.96 ± 0.16 µg/mL) as reference (Figure 2).

**Figure 3:**
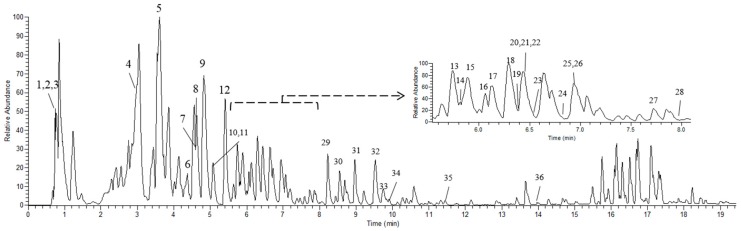
The base peak intensity chromatogram of 70% ethanol solution of the dried ACS extract analyzed by ultra-performance liquid chromatography coupled with electrospray ionization orbitrap mass spectrometry (UPLC-ESI-Orbitrap-MS) in negative ionization mode.

In addition, 36 components including amino acids, phenolic and its glycosides, and flavonoids and its glycosides were tentatively identified by UPLC-ESI-Orbitrap-MS after comparison of the retention time, accurate m/z and mass fragmentation pattern with authentic standards, ACS literatures [6,24,25] as well as online database. The base peak chromatogram of extract of ACS was depicted in Figure 3 and a summary of phytochemicals detected by UPLC-ESI-Orbitrap-MS was shown in Table 1.

### 3.2. Effect on Serum Lipid Profiles

**Figure 4:**
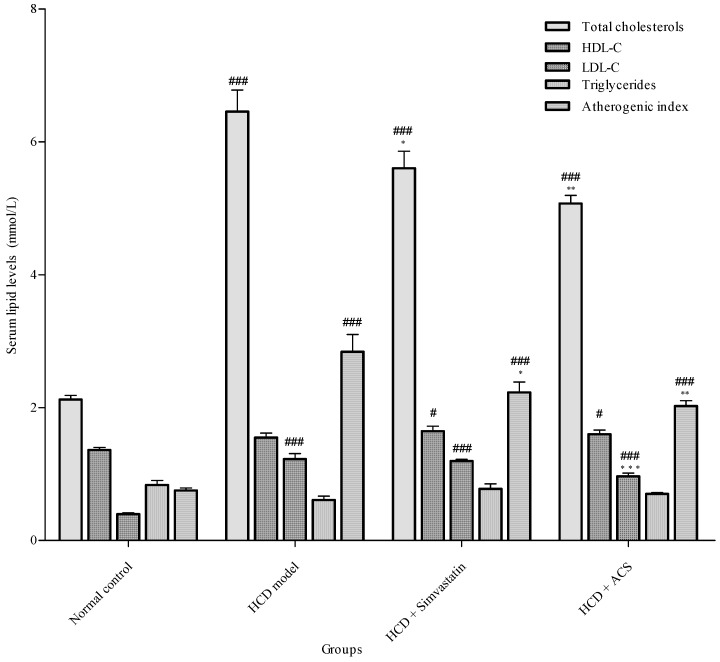
Serum lipid levels and atherogenic indexes of rats. Data are expressed as means ± SEM, (*n* = 5–7). One-way analysis of variance (ANOVA), Tukey HSD post hoc test. # *p* < 0.05 and ### *p* < 0.001 represent significant differences when compared with the normal control group. * *p* < 0.05, ** *p* < 0.01, and *** *p* < 0.001 represent significant differences when compared with the high cholesterol diet (HCD) group.

The serum lipid profiles of different treatment groups by the end of the experiment were summarized in Figure 4. In rats fed with HCD, there were significant elevations in total serum cholesterol (*p* < 0.001), LDL-C (*p* < 0.001) and atherogenic index (*p* < 0.001), as compared with the control group. Treating the animals with ACS extract could suppress the increased serum total cholesterol (*p* < 0.01) and LDL-C (*p* < 0.001) induced by the HCD. However, ACS extracts used did not return the levels of serum total cholesterol and LDL-C back to normal (those of the control group). Both the ACS extract (*p* < 0.01) and simvastatin (*p* < 0.05) supplementation could significantly decrease the atherogenic index which is a parameter to measure the risk of coronary heart disease.

### 3.3. Stability of Metabolomics Platform

The stability of GC-MS detection was assessed by multiple injections of the same pooled sample after extraction at room temperature within 24 h. The coefficient of variation of EFAs and FFAs were 4.04–11.69% and 4.57–13.54%, respectively. Recovery (*n* = 5) by spiking EC18:1n-9c and C18:1n-9c (the same concentration as serum) to the serum was also performed and the obtained results were 95.31 ± 2.87% and 93.08 ± 3.47% (mean ± relative standard deviation).

### 3.4. Effects on Metabolite Changes

**Figure 5:**
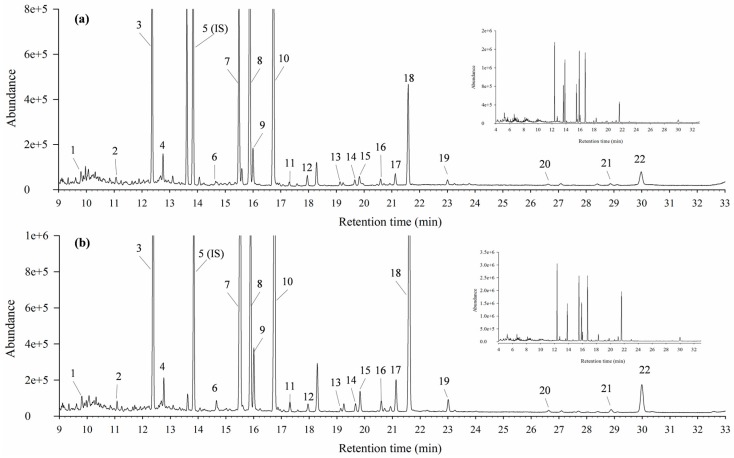
Typical total ion chromatograms of rat serums from HCD group by gas chromatography–mass spectrometry (GC-MS): (**a**) free fatty acids (FFAs) and (**b**) esterified fatty acids (EFAs). IS, internal standard.

**Figure 6:**
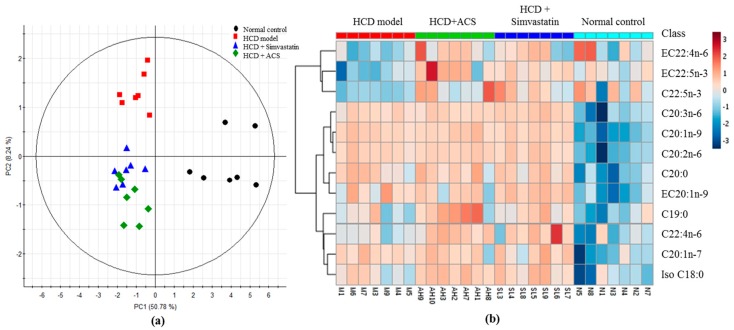
GC-MS results of FFAs and EFAs of rat serum: (**a**) the partial least squares discriminant analysis (PLS-DA) score plot of FFAs and EFAs level data sets (after automatic transformation). R^2^Y (cum) = 0.57, Q^2^ (cum) 0.50; and (**b**) A heatmap of FFAs and EFAs of GC-MS contributing to the classification of PLS-DA [Variable Importance in the Projection (VIP) value ≥ 1].

Total of 21 EFA and 21 FFA were identified in the serum samples by GC-MS (Table 2 and Figure 5). Obvious separation trend among the control, HCD, simvastatin treatment and ACS treatment groups was observed in partial least squares-discrimination analyses (PLS-DAs) being applied (Figure 6a). The cumulative R^2^Y and Q^2^ were 0.57 and 0.50, respectively, and no overfitting was observed.

For the multivariate analysis using PLS-DA, VIP was chosen to find out those peaks in GC-MS data with VIP value ≥ 1. After screening, 12 differential metabolites were further evaluated and Figure 6b clearly visualizes the relative content of differential EFAs and FFAs with VIP ≥ 1 in different groups to analyze their hierarchical cluster by heat map. Esterified 11-eicosenoic acid (EC20:1n-9) and nine FFAs were higher in all three HCD-fed groups while free docosapentaenoic acid (DPA) (C22:5n-3), esterified adrenic acid (EC22:4n-6) and esterified DPA were only increased in both simvastatin and ACS treated groups compared with HCD group (*p* < 0.05).

**Figure 7:**
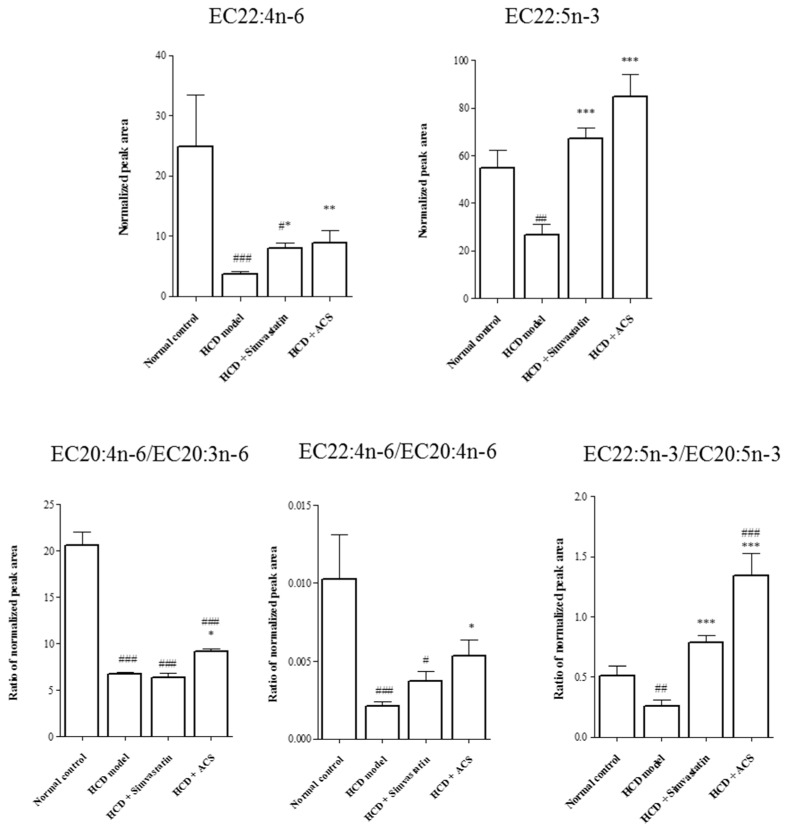
The normalized serum levels of EC22:4n-6, EC22:5n-3, and ratio of EC20:4n-6/EC20:3n-6, EC22:4n-6/EC20:4n-6 and EC22:5n-3/EC20:5n-3. Data are expressed as means ± SEM, (*n* = 6–8). One-way ANOVA test after log 2 transformation to minimize variance, Tukey HSD post hoc test. # *p* < 0.05, ## *p* < 0.01 and ### *p* < 0.001 represent significant differences when compared with the normal control group. * *p* < 0.05, ** *p* < 0.01 and *** *p* < 0.001 represent significant differences when compared with the HCD group.

Figure 7 shows the normalized serum levels of EC22:4n-6 and EC22:5n-3, the ratio of esterified arachidonic acid to esterified dihomo-γ-linolenic acid (EC20:4n-6/EC20:3n-6), the ratio of esterified adrenic acid to esterified arachidonic acid (EC22:4n-6/EC20:4n-6), and the ratio of esterified DPA to esterified eicosapentaenoic acid (EPA) (EC22:5n-3/EC20:5n-3) among normal control, HCD model, HCD treatment with simvastatin and ACS groups. There were significantly decreased level of DPA, adrenic acid, ratio of EC20:4n-6/EC20:3n-6, EC22:4n-6/EC20:4n-6 and EC22:5n-3/EC20:5n-3 in HCD model group. However, treatment with ACS could upregulate all (*p* < 0.05) compared with HCD group.

### 3.5. Effects on the Protein Expression of PPARα in the Liver

**Figure 8:**
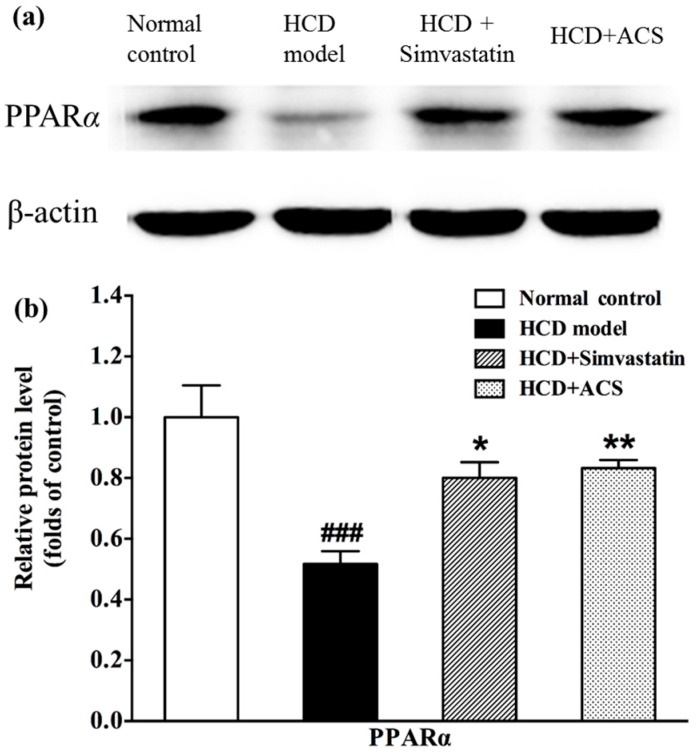
Effects of ACS extract on protein expression of hepatic PPARα of rats: (**a**) protein expression of PPARα in the liver of rats was changed by simvastatin and ACS; and (**b**) the protein levels were expressed relative to β-actin. Data are expressed as means ± SEM (*n* = 7). One-way ANOVA, Tukey HSD post hoc test. ### *p* < 0.001 represent significant differences when compared with the Control group. * *p* < 0.05 and ** *p* < 0.01 represent significant differences when compared with the HCD group.

From the results shown in Figure 8, HCD significantly decreased the PPARα protein level (*p* < 0.001). Interestingly, treating the rats with ACS (*p* < 0.01) and simvastatin (*p* < 0.05) could restore the protein expression of PPAR*α* close to the control under the same experimental conditions.

## 4. Discussion

Diet plays an important role in the control of cholesterol homeostasis and the main reason leading to the development of hyperlipidemia, atherosclerosis and other CVDs because of the consumption of cholesterol-enriched diet over long period of time [26]. TCMs possessing hypocholesterolemic and/or hypolipidemic properties are commonly used as functional foods to prevent the development of CVDs in the Chinese community [27,28,29]. The present study showed that ACS extract supplementation at 600 mg/kg/day for four weeks resulted in suppression of diet-induced hypercholesterolaemia, evidenced by the reduction of serum total cholesterols, LDL-C and atherogenic index, similar to the group given by the positive control, simvastatin.

The pharmacological activities of ACS have been focused and its flavonoid fraction was proved as active constituents. In the present study, the total phenolic content of dried ACS extract was determined by Folin–Ciocalteu method and its chemical constituents of dried ACS were further examined by mass spectrometry. ACS exhibited antioxidant DPPH radical scavenging activity. Multiclass compounds (a total of 36 compounds) including amino acid, flavonoids and their glycosides were identified (eight compounds were identified with standards) or tentatively identified. Some of these compounds have been reported to possess different biological activities. For example, isoquercitrin had a protective effect against acetaminophen induced liver injury [30]; complanatuside exhibited strong inhibition on lipopolysaccharide-induced nitric oxide release by macrophages [31]; and quercetin demonstrated a protective effect on high-fat diet-induced non-alcoholic fatty liver disease in mice mediated by modulating intestinal microbiota imbalance and related gut-liver axis activation [32].

Metabolomics, i.e., systematic analysis of all metabolites and metabolic pathways in a given biological system, has been increasingly recognized in biomarker discovery and understanding disease mechanisms. Good recovery and high analytical reproducibility of the metabolite detection in this study demonstrated that the presented methodologies have the reliability and robustness as required by a metabolomics profiling study. As a result, it could ensure the changes among the different groups observed from the statistical analysis were biologically related.

In the result of GC-MS of EFA and FFA of serum of rat with HCD, it was obvious that the level of DPA and adrenic acid decreased significantly. Dihomo-γ-linolenic acid and arachidonic acid are key players in the synthetic pathway for pro-inflammatory series 2 prostaglandins and leukotrienes and elevated levels of those polyunsaturated fatty acid (PUFA) may contribute to the inflammatory phenotype in obesity or metabolic syndrome [33,34]. The obtained results indicated that suppression on transformation from dihomo-γ-linolenic acid and arachidonic acid to adrenic acid was presented in rat fed with HCD. Based on the route of biosynthesis of the ω-6 family of PUFA [35], the following increased product possibly resulted in up-regulation of pro-inflammatory eicosanoids synthesis. In the opposite, the result of treatment of ACS showed that adrenic acid, ratio of arachidonic acid to dihomo-γ-linolenic acid and ratio of adrenic acid to arachidonic acid were up-regulated significantly (*p* < 0.05). In addition, DPA and its ratio to EPA were elevated significantly (*p* < 0.001), which was probably transformed to more anti-inflammatory eicosanoids in the pathway of biosynthesis of ω-3 PUFA [35].

PPARs are members of the nuclear receptor superfamily of ligand-binding transcription factors that control the genes regulation involved in lipid and glucose metabolism linked to energy homeostasis, adipogenesis, inflammatory responses [36]. Based on the results obtained in GC-MS metabolomics platform, the protein expression of PPARα, which is expressed primarily in the liver, was further investigated on the effect of β-oxidation of fatty acid after ACS treatment. The present study found that the protein expression of PPARα of the treatment group with ACS was upregulated significantly. This implied that the β-oxidation of fatty acids were enhanced. Archana Mishra’s study demonstrated that oxidized ω-3 PUFA inhibited pro-inflammatory responses such as leukocyte adhesion receptor and chemokine expression through inhibition of NF-κB via a PPAR-dependent pathway [37]. In ACS treated group, the increased ω-3 PUFA, and free and esterified DPA that was finally converted to DHA (free or esterified DHA in this study did not have significant difference between groups) was probably inhibited pro-inflammatory responses by oxidation via the same PPARα-dependent mechanism. Thus, a detail study on the gene and protein expression involved in fatty acid oxidation at liver such as carnitine palmitoyltransferase I (CPT1) or acyl-CoA oxidase 1 (ACOX1) is needed for further investigation to consolidate the mechanism behind on the treatment of ACS. 

## 5. Conclusions

The dried 70% ethanol extract isolated from ACS contains rich antioxidant phenolic compounds, which was further identified mainly as flavonoids and flavonoid glycosides by UPLC-ESI-Orbitrap-MS. The present results demonstrated that the ACS significantly reduced the serum total cholesterols and LDL-C in the rats fed with HCD after four-week treatment. Fatty acid profiling analysis also pointed out that DPA in serum of treatment group with ACS has been restored back to normal. One of the hypocholesterolemic activities is associated with its promotion of the β-oxidation of fatty acids in the liver, as demonstrated by the increased protein expressions of PPARα.

## Figures and Tables

**Table 1:** Identification of metabolites from 70% ethanol extract of ACS analyzed by UPLC-ESI-Orbitrap-MS

Peak Number	Retention Time (min)	Identity (Common Name)	Theoretical m/z	Detected m/z	Mass Error (ppm)	Adduct	Molecular Formula	Mass Fragments
1	0.72	l-Arginine *	173.1044	173.1042	−1.4	[M−H]^−^	C_6_H_14_N_4_O_2_	131.0823, 156.0773
2	0.76	l-Aspartic acid *	132.0302	132.0298	−2.7	[M−H]^−^	C_4_H_7_NO_4_	115.0032
3	0.77	l-Glutamic acid *	146.0459	146.0456	−2.2	[M−H]^−^	C_5_H_9_NO_4_	128.0348
4	2.90	Myricetin 3,4′-diglucopyranoside (Complanatoside A)	641.1359	641.1358	−0.2	[M−H]^−^	C_27_H_30_O_18_	317.0296, 479.0825, 623.1239
5	3.61	Myricetin-3*-O-*β-d-glucopyranoside isomer	479.0831	479.0833	0.5	[M−H]^−^	C_21_H_20_O_13_	151.0031, 178.9981, 316.02197
6	4.37	Calycosin 7*-O-*β-d-glucopyranoside/Calycosin 7-β-d-galactopyranoside	491.1195	491.1193	−0.4	[M+HCOO]^−^	C_22_H_22_O_10_	268.0375, 283.0611, 329.1140, 447.1292
7	4.58	Quercetin-3*-O-*β-d-glucopyranoside (Isoquercitrin) *	463.0882	463.0881	−0.2	[M−H]^−^	C_21_H_20_O_12_	151.0031, 178.9981, 271.0244, 300.0271
8	4.63	Laricitrin 3*-O-*β-d-glucopyranoside/Myricomplanoside	493.0988	493.0987	−0.3	[M−H]^−^	C_22_H_22_O_13_	151.0031, 178.9981, 315.0142, 330.0377
9	4.82	Myricetin-3*-O-*β-d-glucopyranoside/Myricetin 5′*-O-*β-d-glucopyranoside/isomer	479.0831	479.0831	0.0	[M−H]^−^	C_21_H_20_O_13_	151.0031, 178.9981, 299.0190, 317.0298
10	5.07	Kaempferol 3*-O-*β-rutinoside (Nicotiflorin)/Kaempferol 3-neohesperidoside/isomer	593.1512	593.1510	−0.3	[M−H]^−^	C_27_H_30_O_15_	151.0032, 178.9982, 285.0400, 447.1192
11	5.07	Quercetin 3*-O-*α-l-arabinopyranoside	433.0776	433.0774	−0.4	[M−H]^−^	C_20_H_18_O_11_	151.0031, 178.9982, 255.0294, 271.0244, 300.271
12	5.41	Kaempferol 3*-O-*β-d-glucopyranoside (Astragalin)	447.0933	447.0933	−0.1	[M−H]^−^	C_21_H_20_O_11_	227.0344, 255.0295,284.0322
13	5.74	Kaempferol 3*-O-*α-l-arabinopyranoside	417.0827	417.0827	−0.1	[M−H]^−^	C_20_H_18_O_10_	151.0032, 178.9980, 227.0344, 255.0294, 284.0322
14	5.84	Kaempferide/Rhamnocitrin	299.0561	299.0557	−1.2	[M−H]^−^	C_16_H_12_O_6_	271.0247, 284.0322
15	5.88	Calycosin	283.0612	283.0611	−0.4	[M−H]^−^	C_16_H_12_O_5_	211.0396, 224.0474, 268.0374
16	6.06	Myricetin	317.0303	317.0299	−1.3	[M−H]^−^	C_15_H_10_O_8_	151.0032, 165.0189, 178.9981, 271.0244, 289.0244, 299.0195
17	6.12	Kaempferol 3*-O-*β-rutinoside isomer	593.1512	593.1510	−0.4	[M−H]^−^	C_27_H_30_O_15_	283.0245, 298.0480
18	6.29	Laricitrin 3*-O-*β-d-glucopyranoside/Myricomplanoside	493.0988	493.0987	−0.1	[M−H]^−^	C_22_H_22_O_13_	151.0031, 178.998, 316.0219, 331.0455
19	6.39	Quercetin-4′*-O-*β-d-glucopyranoside/6-Hydroxykaempferol 7-glucopyranoside/isomer	463.0882	463.0879	−0.7	[M−H]^−^	C_21_H_20_O_12_	151.0031,178.9981, 301.0349
20	6.44	Rhamnocitrin-3*-O-*β-d-glucopyranoside/Kaempferide 7*-O-*β-d-glucopyranoside	461.1089	461.1088	−0.2	[M−H]^−^	C_22_H_22_O_11_	165.0189, 271.0609, 299.0557, 341.0662
21	6.44	Rhamnocitrin-3,4′*-O-*diglucopyranoside (Complanatuside) *	669.1672	669.1673	0.1	[M+HCOO]^−^	C_28_H_32_O_16_	299.0557, 461.1085
22	6.45		623.1618	623.1617	−0.1	[M−H]^−^	C_28_H_32_O_16_	299.0559, 461.1082
23	6.51	Calycosin 7-β-d-glucopyranoside/Calycosin 7-galactopyranoside	445.1140	445.1137	−0.7	[M−H]^−^	C_22_H_22_O_10_	283.0606
24	6.82	Calycosin 7-β-d-glucopyranoside/Calycosin 7-galactopyranoside	445.1140	445.1137	−0.6	[M−H]^−^	C_22_H_22_O_10_	283.0606
25	6.94	Rhamnocitrin-3*-O-*β-d-glucopyranoside/Kaempferide 7*-O-*β-d-glucopyranoside	461.1089	461.1087	−0.4	[M−H]^−^	C_22_H_22_O_11_	165.0187, 299.0564
26	6.94	Neocomplanoside/6″*-O-*acetyl-kaempferide-7*-O-*β-d-glucopyranoside/6″*-O-*acetyl-pratensein-7*-O-*β-d-glucopyranoside	503.1195	503.1193	−0.4	[M−H]^−^	C_24_H_24_O_12_	283.0245, 299.0555, 461.1088
27	7.72	Calycosin 7-β-d-glucopyranoside/Calycosin 7-galactoside	445.114	445.1137	−0.7	[M−H]^−^	C_22_H_22_O_10_	283.0606
28	7.97	Quercetin *	301.0350	301.0350	−1.3	[M−H]^−^	C_15_H_10_O_7_	121.0290, 151.0032, 178.9981, 273.0403
29	8.22	Kaempferol 3*-O-*β-rutinoside isomer	593.1512	593.1513	0.1	[M−H]^−^	C_27_H_30_O_15_	283.0243, 298.0478
30	8.55	Formononetin 7*-O-*(6″-acetylglucoside)/ isomer	517.1351	517.1351	0.0	[M+HCOO]^−^	C_24_H_24_O_10_	252.0422, 267.0659
31	8.97	Rhamnocitrin-3*-O-*β-d-glucopyranoside/Kaempferide 7*-O-*β-d-glucopyranoside	461.1089	461.1090	0.3	[M−H]^−^	C_22_H_22_O_11_	299.0555
32	9.53	Neocomplanoside/6″*-O-*acetyl-kaempferide-7*-O-*β-d-glucoside/6″*-O-*acetyl-pratensein-7*-O-*β-d-glucoside	503.1195	503.1194	−0.1	[M−H]^−^	C_24_H_24_O_12_	283.0244, 299.0555, 461.1096
33	9.72	Kaempferol *	285.0405	285.0405	−0.1	[M−H]^−^	C_15_H_10_O_6_	185.0602, 229.0514, 257.0457
34	9.89	Formononetin 7*-O-*(6″-acetylglucoside)/isomer	517.1351	517.1348	−0.6	[M+HCOO]^−^	C_24_H_24_O_10_	252.0423, 267.0659
35	11.48	Formononetin *	267.0663	267.0663	0.0	[M−H]^−^	C_16_H_12_O_4_	223.0397, 252.0424
36	13.96	Kaempferide/Rhamnocitrin/Isokaempferide	299.0561	299.0559	−0.6	[M−H]^−^	C_16_H_12_O_6_	165.0188, 271.0608, 284.0321

* Peaks were identified by standards. Others were tentatively identified with references and online database.

**Table 2:** Identification of FFAs and EFAs from the rat serum analyzed by GC-MS.

Peak Number	Retention Time (min)	Abbreviation		Identity (Common Name)	Molecular Formula
		FFA	EFA		
1	9.97	C14:0	EC14:0	Methyl n-tetradecanoate (Myristic acid methyl ester)	C_15_H_30_O_2_
2	11.08	C15:0	EC15:0	Methyl n-pentadecanoate	C_16_H_32_O_2_
3	12.37	C16:0	EC16:0	methyl hexadecanoate (Palmitic acid methyl ester)	C_17_H_34_O_2_
4	12.77	C16:1n-7	EC16:1n-7	Methyl cis-9-hexadecenoate (Palmitoleic acid methyl ester)	C_17_H_32_O_2_
5 (internal standard)	13.84	C17:0	EC17:0	Methyl heptadecanoate	C_18_H_36_O_2_
6	14.66	iso C18:0	iso EC18:0	Methyl 16-methylheptadecanoate (Iso-stearic acid methyl ester)	C_19_H_38_O_2_
7	15.49	C18:0	EC18:0	Methyl octadecanoate (Stearic acid methyl ester)	C_19_H_38_O_2_
8	15.89	C18:1n-9	EC18:1n-9	Methyl cis-9-octadecenoate (Oleic acid methyl ester)	C_19_H_36_O_2_
9	16.01	C18:1n-7	EC18:1n-7	Methyl cis-11-octadecenoate (cis-Vaccenic acid methyl ester)	C_19_H_36_O_2_
10	16.74	C18:2n-6	EC18:2n-6	Methyl cis-9,12-octadecadienoate (Linoleic acid methyl ester)	C_19_H_34_O_2_
11	17.30	C19:0	EC19:0	Methyl nonadecanoate	C_20_H_40_O_2_
12	17.96	C18:3n-3	EC18:3n-3	Methyl all-cis-9,12,15-octadecatrienoate (α-Linolenic acid methyl ester)	C_19_H_32_O_2_
13	19.24	C20:0	EC20:0	Methyl eicosanoate (Arachidic acid methyl ester)	C_21_H_42_O_2_
14	19.67	C20:1n-9	EC20:1n-9	Methyl cis-11-eicosenoate	C_21_H_40_O_2_
15	19.83	C20:1n-7	EC20:1n-7	Methyl cis-13-eicosenoate	C_21_H_40_O_2_
16	20.60	C20:2n-6	EC20:2n-6	Methyl cis-11,14-eicosadienoate	C_21_H_38_O_2_
17	21.13	C20:3n-6	EC20:3n-6	Methyl cis-8,11,14-eicosatrienoate (Dihomo-γ-linolenic acid methyl ester)	C_21_H_36_O_2_
18	21.59	C20:4n-6	EC20:4n-6	Mehyl cis-5,8,11,14-eicosatetraenoate (Arachidonic acid methyl ester)	C_21_H_34_O_2_
19	23.01	C20:5n-3	EC20:5n-3	methyl cis-5,8,11,14,17-eicosapentaenoate	C_21_H_32_O_2_
20	26.64	C22:4n-6	EC22:4n-6	methyl cis-7,10,13,16-docosatetraenoate (Adrenic acid methyl ester)	C_23_H_38_O_2_
21	28.88	C22:5n-3	EC22:5n-3	methyl cis-7,10,13,16,19-docosapentaenoate	C_23_H_36_O_2_
22	29.98	C22:6n-3	EC22:6n-3	methyl cis-4,7,10,13,16,19-docosahexaenoate	C_23_H_34_O_2_
